# GDF-15 levels in patients with polycystic ovary syndrome treated with metformin: a combined clinical and in silico pathway analysis

**DOI:** 10.20945/2359-4292-2023-0416

**Published:** 2024-08-27

**Authors:** Fernanda M. V. Magalhães, Rodrigo M. C. Pestana, Cláudia N. Ferreira, Ieda F. O. Silva, Ana L. Candido, Flávia R. Oliveira, Fernando M. Reis, Karina B. Gomes

**Affiliations:** 1 Faculdade de Farmácia Universidade Federal de Minas Gerais Belo Horizonte MG Brasil Faculdade de Farmácia, Universidade Federal de Minas Gerais, Belo Horizonte, MG, Brasil; 2 Faculdade de Medicina Universidade Federal de Minas Gerais Belo Horizonte MG Brasil Faculdade de Medicina, Universidade Federal de Minas Gerais, Belo Horizonte, MG, Brasil; 3 Colégio Técnico Universidade Federal de Minas Gerais Belo Horizonte MG Brasil Colégio Técnico, Universidade Federal de Minas Gerais, Belo Horizonte, MG, Brasil

**Keywords:** GDF-15, polycystic ovary syndrome, metformin, biologic pathways

## Abstract

**Objective:**

Polycystic ovary syndrome (PCOS) is an endocrine disease characterized by metabolic, reproductive, and psychological manifestations. Growth and differentiation factor 15 (GDF-15) is a cytokine associated with metabolic and inflammatory disorders. Metformin is commonly used for the treatment of PCOS. We investigated the relationship between GDF-15 levels and PCOS, the effect of metformin on GDF-15 levels, and potential biologic pathways related to GDF-15.

**Subjects and methods:**

The study included 35 women with PCOS and 32 women without PCOS (controls). Both groups were compared in terms of GDF-15 levels. Additional analysis was conducted on samples from 22 women with PCOS who were treated with either metformin (n = 7) or placebo (n = 15), retrieved from a previous randomized, controlled trial. Levels of GDF-15 were measured using MILLIPLEX. The biologic pathways related to GDF-15 were evaluated using the databases STRING, SIGNOR, and Pathway Commons. The statistical analysis was conducted using the software SPSS.

**Results:**

Levels of GDF-15 were higher in the PCOS group compared with the non-PCOS group (p = 0.039). Among women with PCOS, GDF-15 levels were higher in those treated with metformin compared with placebo (p = 0.007). The proteins related to GDF-15 overlapped between the databases, and a significant interaction was found between GDF-15 and proteins related to PCOS and its complications, including those related to estrogen response, oxidative stress, ovarian infertility, interleukin (IL)-18, IL-4, the ratio of advanced glycation end products to their receptor (AGE/RAGE), leptin, transforming growth factor beta (TGF-β), adipogenesis, and insulin.

**Conclusion:**

The findings of the present study suggest a relationship between GDF-15 and PCOS and a potential increase in GDF-15 levels with metformin treatment. An additional finding was that GDF-15 could be involved in biologic pathways related to PCOS complications.

## INTRODUCTION

Polycystic ovary syndrome (PCOS) is considered the most common endocrine disorder in reproductive age, affecting approximately 5%-18% of women in this phase. Due to its current prevalence and associated comorbidities, PCOS is an important public health problem (1,2). The diagnosis of PCOS is based on the presence of at least two of three clinical manifestations, as defined by the Rotterdam criteria: (A) chronic oligo-ovulation or anovulation, (B) clinical and/or laboratory hyperandrogenism, and (C) ultrasonographic characteristics of polycystic ovaries and/or ovarian volume > 10 mL (1,3).

In addition to these core signs and symptoms, PCOS may have many clinical manifestations (4), including precocious puberty, infertility, and pregnancy complications (5,6). Other complications of PCOS include primary insulin resistance on muscle and adipose tissue level with compensatory hyperinsulinemia, intrinsic beta-cell dysfunction, type 2 diabetes mellitus (T2DM), dyslipidemia, nonalcoholic fatty liver disease, increased risk of cardiovascular disease, obesity, and metabolic syndrome (7,8). Psychological manifestations of PCOS are represented mainly by the development of anxiety, depression, and lack of acceptance of body image (3).

Notably, PCOS is also associated with low-grade chronic systemic inflammation (9). Stimuli associated with PCOS activate inflammatory cells (*e.g.*, macrophages) and induce the production of cytokines related to inflammation (10,11). Levels of C-reactive protein (CRP) – produced by hepatocytes in response to stimulation by proinflammatory cytokines such as interleukin (IL)-6 and tumor necrosis factor alpha (TNF-a) – are on average 96% greater in women with PCOS than in healthy controls. Additionally, PCOS is associated with elevation in levels of IL-18, monocyte chemoattractant protein 1 (MCP-1), and macrophage inflammatory protein 1 (MIP-1), which correlate directly with obesity, total testosterone level, and hirsutism and inversely with insulin sensitivity index (11). A study by our group has observed decreased tumor necrosis factor (TNF) to IL-6, TNF to IL-2, and TNF to IL-4 ratios among 97 patients with PCOS compared with 99 controls (12).

Growth and differentiation factor 15 (GDF-15) is a cytokine of the transforming growth factor beta (TGF-β) family that has been increasingly studied for its relationship with several physiological and pathological processes, including stress-induced inflammation, cardiovascular diseases, kidney diseases, and obesity, probably in a counterregulatory way (13-17). In fact, GDF-15 can regulate lipid and glucose metabolism, increasing insulin sensitivity and protecting against chronic inflammation of adipose tissue (18).

Metformin is a biguanide commonly prescribed for the treatment of T2DM and PCOS due to its excellent tolerability, low cost, and safe profile (19-21). Metformin helps restore the body’s response to insulin, decreasing the production of glucose by the liver and the absorption of sugar by the intestine and stomach. In patients with PCOS, evidence shows that metformin also restores ovulation, reduces symptoms linked to hyperandrogenism, regulates menstrual cycles, and increases fertility (20). Recent studies have investigated the relationship between GDF-15 levels and metformin effects, particularly in the context of T2DM, obesity (22,23), postprandial states (24), coronary artery disease, and cancer (25). However, it remains unclear how GDF-15 levels are affected by PCOS and whether metformin treatment alters these levels in patients with this highly prevalent condition. Only a few studies have evaluated the relationship between GDF-15 and PCOS, and these studies have involved patients within a narrow age range (26,27). To the best of our knowledge, no studies to date have analyzed the association between GDF-15 levels and metformin treatment in patients with PCOS, or the possible GDF-15 biologic pathways.

Considering the association of GDF-15 with inflammatory and metabolic diseases, the aim of this study was to explore the hypothesis that GDF-15 may be linked to PCOS and its related complications, which commonly include inflammation and metabolic disorders. Additionally, we investigated whether metformin could regulate GDF-15 production and explored potential biologic pathways through which GDF-15 might influence the pathophysiology of PCOS.

## SUBJECTS AND METHODS

### Subjects

The first part of this study had a cross-sectional design and included 35 women with PCOS (PCOS group) and 32 women without PCOS (non-PCOS group), all of whom were aged 18-40 years.

Patients with PCOS were recruited from the Hyperandrogenism Outpatient Clinic of *Hospital das Clínicas* at Federal University of Minas Gerais (UFMG). The diagnosis of PCOS was established according to diagnostic criteria established by the European Society of Human Reproduction and Embryology (ESHRE)/American Society for Reproductive Medicine (ASRM) PCOS Consensus Workshop Group (28). The diagnosis of PCOS based on these criteria requires at least two of the three following manifestations: (A) oligo-ovulation and/or anovulation, (B) clinical and/or biochemical signs of hyperandrogenism, (C) polycystic ovaries (12 or more follicles in each ovary measuring 2-9 mm in diameter, and/or increased ovarian volume > 10 mL). Participants in the non-PCOS group were recruited from the Gynecological Outpatient Clinic at the same institution. Women in this group had regular ovulatory cycles with regular menses lasting 25-35 days and serum progesterone level > 5 ng/mL during the luteal phase. Additionally, women in this group had normal androgen levels, no hirsutism, and no polycystic ovaries on ultrasound. All participants suspended physical activities 24 hours prior to the study.

We excluded from the study participants in both groups who had a previous diagnosis of T2DM, tumors, inflammatory disease, hyperprolactinemia (prolactin level > 40 ng/mL), hypogonadism, pregnancy, treatment with steroids or nonsteroidal antiinflammatory drugs, isotretinoin, cyclosporine, antiretroviral, insulin, and oral contraceptives, and adrenal, kidney, liver, or thyroid disorders.

The second part of this study assessed the regulation of metformin on GDF-15 levels in women with PCOS using serum samples from a previous randomized, controlled trial (29), which had the same inclusion and exclusion criteria listed above. The participants were randomized to receive a single nightly dose of either placebo (n = 15) or metformin 1,500 mg (Glifage XR, Merck S.A., Rio de Janeiro, Brazil; n = 7) for 60 days. Blood samples for measurement of GDF-15 levels were obtained at the end of treatment.

### Ethics

The protocol of the cross-sectional study was approved by the Ethics Committee of the Federal University of Minas Gerais (CAAE 0379.0.203.000-11). The protocol of the randomized, controlled trial was registered at the Brazilian Clinical Trials Registry, REBEC (Primary ID Number RBR-47tvky) and at the Brazilian Ministry of Health Human Research Registry (Protocol ID 17127713.2.0000.5149). Informed consent was obtained from all participants. The study was conducted in accordance with the Declaration of Helsinki.

### Biochemical determinations

Venous blood samples were collected after a 12-hour fast. These samples were centrifuged at 2,500 g for 20 minutes at 4°C to separate the plasma (using EDTA or citrate) or serum. The resulting aliquots were stored at -80 °C until they underwent analysis.

Fasting blood glucose, total cholesterol, high-density lipoprotein (HDL) cholesterol, triglycerides, and CRP were measured using the VITROS System (Johnson & Johnson, New Brunswick, NJ, USA). Levels of low-density lipoprotein (LDL) cholesterol and very-low-density lipoprotein (VLDL) cholesterol were estimated. Insulin and total testosterone levels were measured using chemiluminescence on the ARCHITECT system (Abbott, Chicago, IL, USA). All measurements were performed according to manufacturers’ instructions.

Participants’ weight was measured with a calibrated scale, height with a rigid stadiometer, and waist circumference with a tape measure. We calculated the lipid accumulation product (LAP) index using the following formula:


$$\begin{array}{cc} LAP= & (\text{ waist circumference in }cm-58)\\ & x\text{ triglycerides }(mmol/L) \end{array}$$

Finally, we calculated the homeostatic model assessment of insulin resistance (HOMA-IR) index using the following formula:


HOMA-IR = (insulin in mIU/Lx fasting glucose in mmol/L)/22.5

### Measurement of GDF-15 levels

Plasma samples were collected in citrate-coated vacuum tubes from participants in the cross-sectional evaluation, whereas serum samples were obtained from the participants in the randomized, controlled trial. The aliquots were stored at -80 °C until analyzed. Levels of GDF-15 were quantified using the kit MILLIPLEX MAP Human Cardiovascular Disease Magnetic Bead Panel 2 (catalog # HCVD2MAG-67K, Millipore, Merck KGaA, Darmstadt, Germany), adhering to the manufacturer’s instructions. The analysis was performed using the MAGPIX system (Luminex Corporation, Austin, TX, USA). The concentrations were determined after fitting the standard curve using the MILLIPLEX Analyst Software. The method has intra-assay and inter-assay coefficients of variation of 3.1% and 11.2%, respectively, and a sensitivity of 0.0006 ng/mL.

### Metabolic pathways

In order to develop an integrated understanding of the proteins related to GDF-15 pathways and PCOS, we applied a network biology approach using the databases SIGNOR (https://signor.uniroma2.it/) and Pathway Commons (https://www.pathwaycommons.org/). The proteins that interact with GDF-15 from both databases overlapped, and the STRING (https://string-db.org/) database showed significant (p < 0.050) biologic pathways related to these interactions.

### Statistical analysis

The statistical analyses were performed using SPSS, Version 21.0 for IOS (IBM Corp., Armonk, NY, USA). The data are presented as mean ± standard deviation or median (interquartile range [IQR]), as appropriate. The normality of quantitative variables was assessed using the Shapiro-Wilk test. Student’s *t* test and Mann-Whitney test were used to determine differences between groups for data with normal and non-normal distribution, respectively. The level of statistical significance was set at a p value < 0.05.

## RESULTS

The clinical and laboratory characteristics of the participants are described in Table 1. The PCOS group, compared with the non-PCOS group, had higher values of insulin, HOMA-IR, CRP, and total testosterone, in addition to greater body mass index, abdominal circumference, and LAP (all p < 0.05). No significant differences in other parameters were observed between the two groups (p > 0.05). The characteristics of the participants in the randomized, controlled trial of metformin are detailed in the corresponding article (26) and presented in Supplementary Material 1.

**Table 1:** Clinical and laboratory variables in the groups with and without polycystic ovary syndrome

Variables	PCOS N = 35	Non-PCOS N = 32	P value
Age (years)	31.00 (5.00)	30.50 (14.00)	0.984
BMI (kg/m^2^)	33.25 (9.54)	22.94 (5.38)	**<0.001**
AC (cm)	106.29 ± 17.85	82.79 ± 14.74	**<0.001**
LAP	65.49 (58.71)	6.13 (32.42)	**<0.001**
Fasting glucose (mmol/L)	85.00 (14.0)	87.00 (13.80)	0.591
HOMA-IR	2.90 (3.49)	1.54 (1.04)	**<0.001**
Insulin (µIU/mL)	14.80 (17.38)	7.30 (7.82)	**<0.001**
TC (mmol/L)	193.31 ± 32.71	183.95 ± 42.48	0.499
HDL-c (mmol/L)	49.05 ± 14.70	57.00 ± 11.31	0.134
LDL-c (mmol/L)	114.50 (32.55)	97.00 (55.75)	0.251
VLDL-c (mmol/L)	21.40 (15.55)	18.50 (10.10)	0.540
TG (mmol/L)	106.00 (77.75)	91.00 (49.50)	0.555
Total testosterone (ng/dL)	61.00 (80.13)	28.50 (26.75)	**0.033**
CRP (mg/dL)	11.00 (12.25)	4.50 (2.00)	**<0.001**

Abbreviations: AC, abdominal circumference; BMI, body mass index; CRP, C-reactive protein; HDL-c, high-density lipoprotein cholesterol; HOMA-IR, homeostatic model assessment of insulin resistance; LAP, lipid accumulation product; LDL-c, low-density lipoprotein cholesterol; PCOS, polycystic ovary syndrome; TG, triglycerides; TC, total cholesterol; VLDL-c, very–low-density lipoprotein cholesterol. P levels < 0.05 were considered significant.

**Figure 1:**
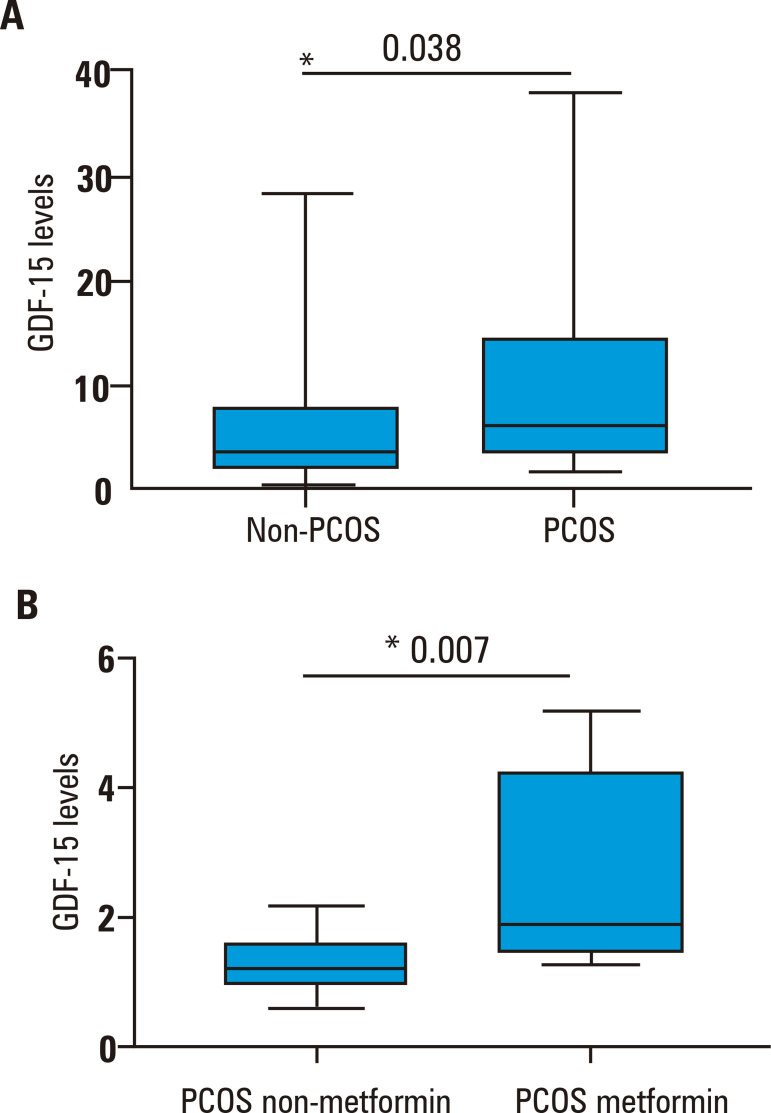
Median levels of growth and differentiation factor 15 (GDF-15; in ng/mL) in women with polycystic ovary syndrome (PCOS group). (A) GDF-15 levels in the PCOS and non-PCOS groups. (B) GDF-15 levels in women with PCOS treated with metformin (PCOS metformin) and not treated with metformin (PCOS non-metformin).

Levels of GDF-15 were higher in the PCOS group (6.11 ng/mL, IQR 10.35 ng/mL) compared with the non-PCOS group (3.62 ng/mL, IQR 5.67 ng/mL; p = 0.039) (Figure 1A). In the PCOS group, GDF-15 levels correlated significantly with VLDL cholesterol (r = 0.457, p = 0.010) and triglycerides (r = 0.451, p = 0.010). Additionally, GDF-15 levels were higher (1.89 ng/mL, IQR 2.73 ng/mL) among women previously treated with metformin compared to those who were never treated with metformin (1.18 ng/mL, IQR 2.73 ng/mL; p = 0.007) in the PCOS group (Figure 1B).

**Figure 2:**
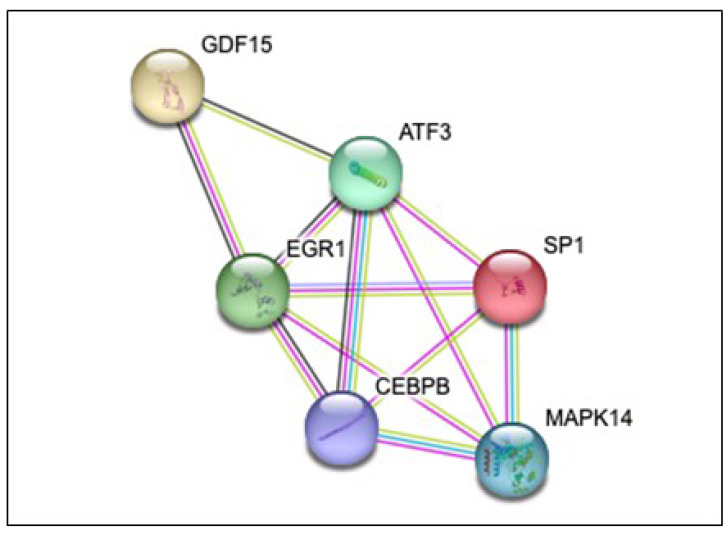
Interactions between proteins and growth and differentiation factor 15 (GDF-15). Protein-protein interactions. Each node represents a protein, and each line refers to an interaction. Line color: blue, known interaction of selected databases; pink, known interaction determined experimentally; green, text mining; black, coexpression. There are 12 potential protein-protein interactions. Enrichment p < 0.0001.

The proteins related to GDF-15 overlapped in the different databases, and we found a significant interaction of GDF-15 with transcription factor SP1 (SP1), MAP kinase-activated protein kinase 14 (MAPK14), early growth response protein 1 (EGR1), CCAAT/enhancer-binding protein beta (CEBPB), and cyclic AMP-dependent transcription factor ATF-3 (ATF3) proteins (p < 0.0001) (Figure 2). The pathways that share common aspects with the pathophysiology of PCOS and its complications and that showed a significant relationship with GDF-15 were estrogen (false discovery rate [FDR] 0.0032), oxidative stress response (FDR 0.0049), ovarian infertility (FDR 0.0049), IL-18 (FDR 0.0049), IL-4 (FDR 0.0081), the ratio of advanced glycation end products (AGEs) to their receptor (RAGE; FDR 0.0109), leptin signaling pathway (FDR 0.0110), TGF-β pathways (FDR 0.014), adipogenesis (FDR 0.0265), and insulin signaling (FDR 0.0335).

## DISCUSSION

The results of the present study showed that women with PCOS had higher GDF-15 levels than women without PCOS, and that treatment with metformin compared with placebo was associated with higher GDF-15 levels. Additionally, GDF-15 levels appeared to be involved in biologic pathways related to inflammation, glucose metabolism, adipogenesis, and infertility, which are pathways also involved in the pathophysiology of PCOS.

The expression of GDF-15 is increased in cardiovascular diseases and metabolic disorders, probably as part of a counterregulatory process intended to reestablish homeostasis (13,16). Previous studies analyzing GDF-15 levels in women with PCOS have found that this protein could be a link to the increased risk of T2DM and cardiovascular disease in PCOS (26). Additionally, studies have reported that GDF-15 has a protective effect against tissue damage (30), plays an important role in the body’s response to insulin (31), and may serve as a marker of cross-regulation between bone and energy metabolism (32). A relative deficit of GDF-15 observed in the early phases of PCOS could partly explain the difficulties that women experience in controlling their body adiposity in this period (27). Additionally, GDF-15 may have antiinflammatory effects (33) and can potentially improve glucose tolerance (34).

In the present study, there was a positive correlation between values of GDF-15 and levels of VLDL cholesterol and triglycerides in the PCOS group, although the levels of these lipids were comparable between the groups. Luan cols. (2019) have shown that GDF-15 stimulates hepatic triglyceride secretion in response to adrenergic signaling, suggesting that GDF-15 coordinates tolerance to inflammatory damage through regulation of triglyceride metabolism (35). However, a higher GDF-15 level in PCOS, in response to the chronic subclinical inflammatory state observed in this disorder, could result in higher triglyceride levels and, consequently, increased cardiovascular risk in women with PCOS.

The GDF-15 levels were higher in patients with PCOS who received metformin compared with placebo. This finding is aligned with the results of an observational study that reported that GDF-15 levels increased with metformin treatment in a dose-dependent manner in individuals with dysglycemia, indicating that GDF-15 could be used as a new biomarker to monitor the efficacy of metformin treatment (36). Considering that GDF-15 not only improves body mass index, fasting insulin levels, and glucose tolerance but may also offer a protective effect against inflammation, we can hypothesize that metformin might address these metabolic complications in PCOS through the action of GDF-15.

In another study evaluating the relationship between GDF-15, use of metformin, and body weight loss, the authors questioned whether weight loss influenced by GDF-15 contributes to enhanced insulin sensitivity (37). Coll and cols. (2020) reported that the use of metformin for 2 weeks increased circulating GDF-15 levels by about 2.5 times in a clinical study. Using a mouse model, the authors demonstrated in the same study that metformin prevented weight gain in response to a high-fat diet, which was attributed to high circulating levels of GDF-15, indicating that this protein has beneficial effects on energy balance and body weight (22).

In the *in silico* analysis in the present study, GDF-15 showed interaction with SP1, MAPK14, EGR1, CEBPB, and ATF3 proteins, which are related to various signaling pathways, including estrogen, oxidative stress, IL-18, IL-4, AGE/RAGE, leptin, TGF-β, and mechanisms controlling adipogenesis and insulin sensitivity.

Only a few studies have evaluated the relationship between androgens, infertility, and GDF-15 levels. In a study by Faubion cols. (2020) in men with coronary artery disease, GDF-15 levels correlated inversely with serum testosterone levels and testosterone-to-estradiol ratio. In addition, protein expression was reduced in the presence of estradiol, which may support the hypothesis of the relationship of GDF-15 with ovarian cycle dysfunction (38). The follicular fluid is a microenvironment for oocyte development and maturation, since ovarian folliculogenesis is regulated by extra-ovarian and intra-ovarian factors. Souček cols. (2018) evaluated the concentration of GDF-15 in the follicular fluid and fertility status in 26 women undergoing assisted reproduction procedures (39). The authors found that GDF-15 levels were not associated with fertility status; however, the granulosa cells were found to secrete GDF-15 into culture media, and primary oocytes displayed cytoplasmic GDF-15 positivity in immunostained ovaries (39). Therefore, in women with PCOS, increased GDF-15 levels could reduce hyperandrogenism – the main characteristic of the disease – but the GDF-15 effects on ovulation should be further investigated.

Notably, GDF-15 is related to oxidative stress and inflammation. Increased GDF-15 levels may be mediated by mitochondrial stress, with activation of the AMP-activated protein kinase (AMPK) pathway (33). However, a study analyzing several inflammatory markers found that IL-18, but not GDF-15, was related to T2DM progression, with a deleterious effect on insulin production or action (40). Some studies have correlated increased GDF-15 levels with increased IL-4 levels and insulin resistance (41,42). The AGE/RAGE axis, one of the GDF-15-related signaling pathways identified in the present study, was inhibited by increased expression of GDF-15, which has also been found to inhibit renal inflammation in patients with diabetic nephropathy (43). These findings suggest that GDF-15 could reduce the damage induced by oxidative stress related to the activation of the AGE pathway on different tissues. Higher GDF-15 levels may also be associated with an antiinflammatory counterregulatory effect, which improves insulin sensitivity and reduces the risk of T2DM development in PCOS.

Evidence shows that GDF-15 is a prognostic biomarker of metabolic disorders related to adiposity and obesity (44,45). Leptin – a protein synthesized by adipocytes – acts directly on the hypothalamus, reducing food intake and increasing energy expenditure. Kralisch cols. (2020) observed increased GDF-15 levels in mice with lipodystrophy, which decreased after treatment with leptin (46). The authors also observed that GDF-15 levels increased during physical exercise and that this effect was regulated by glucagon-insulin interactions (46). Higher GDF-15 levels were also associated with reduced body weight and improved blood glucose levels, mediated by an enhanced feeling of satiety. This effect is facilitated through the central intracellular signaling complex GDF-15, glial cell-derived neurotrophic factor family receptor α-like (GFRAL), and receptor tyrosine kinase (RET). These mechanisms result in decreased hyperinsulinemia, greater hepatic sensitivity to insulin, and, to a lesser extent, greater peripheral sensitivity to insulin, since insulin increases the use of glucose by the muscle (23,47). Based on the findings of our study, treatment with metformin increases GDF-15 levels, which may lead to decreased body weight and improved insulin signaling. These effects could help lower increased blood glucose levels and cardiovascular risk in women with PCOS.

This study has some limitations. First, it enrolled patients from a single center, which may restrict the generalizability of the findings to the Brazilian population alone. Second, the study did not categorize women with PCOS into distinct disease phenotypes. Since GDF-15 may behave differently across various PCOS subtypes, this lack of classification could affect the generalizability and precision of the results. Third, the sample size precluded us from analyzing the correlation between the participants’ GDF-15 levels and metformin dose. Therefore, an expansion of the study is necessary for a better understanding of the relationship between GDF-15 levels and metformin dose. Fourth, the GDF-15 levels were lower in the set of samples obtained from the randomized, controlled trial. This variation may have been caused by the different methods used for blood processing of the samples, specifically plasma versus serum. This finding suggests that the methodology is sensitive to the type of blood sample, and a cross-validation of the results between the two groups of samples was not possible. Consequently, a replication of these results must carefully consider preanalytical variables.

An important strength of our study is that, to our knowledge, this is the first case-control study examining GDF-15 levels in women with PCOS taking into consideration the use of metformin. This is also the first study to propose possible biologic pathways through which GDF-15 may be involved in PCOS and its complications.

In conclusion, the results of the present study suggest a relationship between GDF-15 and PCOS and a potential effect of metformin in increasing GDF-15 levels. To our knowledge, this study is the first to analyze GDF-15 levels in women with PCOS across a range of ages. Finally, GDF-15 is involved in biologic pathways related to inflammation, glucose metabolism, adipogenesis, and infertility, all of which are related to PCOS complications.
